# Piezo2 protein: A novel regulator of tumor angiogenesis and hyperpermeability

**DOI:** 10.18632/oncotarget.10134

**Published:** 2016-06-17

**Authors:** Hong Yang, Chang Liu, Rong-Mei Zhou, Jin Yao, Xiu-Miao Li, Yi Shen, Hong Cheng, Jun Yuan, Biao Yan, Qin Jiang

**Affiliations:** ^1^ Eye Hospital, Nanjing Medical University, Nanjing, China; ^2^ The Fourth School of Clinical Medicine, Nanjing Medical University, Nanjing, China; ^3^ Department of Neurology, Jiangsu Province Hospital, Nanjing, China; ^4^ Department of Neurology, Jiangsu Chinese Medicine Hospital, Nanjing, China

**Keywords:** tumor angiogenesis, Piezo2, vascular permeability, endothelial cell

## Abstract

Angiogenesis is important for invasive tumor growth and metastasis. Its inhibition is a promising tactic for limiting tumor progression. Here, we showed that Piezo2 knockdown led to decreased glioma angiogenesis and reduced vascular hyperpermeability. Piezo2 was highly expressed in tumor endothelial cells, and its knockdown suppressed vascular leakage and tumor angiogenesis. In a retinal vasculature development assay, corneal angiogenesis assay and a modified Miles assay, Piezo2 knockdown obviously decreased angiogenesis and vascular hyperpermeability. *In vitro* assays revealed that Piezo2 knockdown inhibited endothelial cell proliferation, migration, and tube formation. Moreover, *In vitro* co-culture system assay showed that Piezo2 knockdown in endothelial cells suppressed cell proliferation, migration, and invasion of glioma tumor cells. Piezo2 could regulate glioma angiogenesis via Ca^2+^/Wnt11/β-catenin signaling in endothelial cells. Taken together, these studies provide the evidence for Piezo2 as a critical regulator of tumor angiogenesis and vascular permeability.

## INTRODUCTION

Angiogenesis, the growth of new blood vessels from preexisting vasculature, is important for tumor growth and metastasis. Tumor neovessels are involved in managing O_2_, nutrient supply, and the clearance of CO_2_ and metabolite in tumor tissue [1, 2]. Tumor vasculature is also one of the important routes of tumor cell metastasis to distant organs. Thus, controlling tumor angiogenesis is a promising tactic in limiting tumor growth and metastasis [3]. Increasing attempts have been conducted to develop various angiogenesis-inhibiting agents. The agents targeting vascular endothelial growth factor (VEGF) and its receptors have been proved to be effective in clinical practice [4]. Moreover, ongoing drug development has focused on regulating other angiogenic pathways [5]. However, current angiogenesis-inhibiting agents are usually cytostatic and only target newly growing tumor vasculature. In addition, they can produce adverse effects such as hypertension, proteinuria, and hemorrhage [6]. Thus, it is still required to further understanding the mechanism of tumor angiogenesis to develop more selective and potent anti-tumor strategies.

Piezo proteins are ion channels, which have been reported to regulate mechanosensory transduction in mammalian cells [7]. They play great roles in a variety of biological processes, such as sensing touch/pain (somatosensation), sound (hearing), and shear stress (cardiovascular physiology) [8, 9]. Piezo mutants have been reported in several human disorders, including hereditary xerocytosis and several syndromes with muscular contracture [10]. Piezo2 protein, encoded by Piezo2/FAM38B genes, was initially identified as mechanically activated (MA) ion channels in the murine neuroblastoma cell line N2A. Piezo2 participates in recognition of light touch and noxious stimuli touch sensation, inflammatory response, mechanical allodynia, and several cellular processes such as proliferation, differentiation, and migration [11–13]. Tumor angiogenesis is associated with many biological processes, including cell proliferation, cell motility, immune, and inflammatory response. Inspired by these findings, we speculated that Piezo2 is a potential regulator of tumor angiogenesis. However, the expression and function of Piezo2 in tumor angiogenesis has not been elucidated.

In this study, we investigated the role of Piezo2 in glioma tumor progression. We showed that Piezo2 knockdown suppressed tumor angiogenesis and vascular permeability *in vivo*. Piezo2 regulated endothelial cell proliferation, migration, and tube formation *In vitro*. Thus, Piezo2 is a potential therapeutic target for tumor angiogenesis.

## RESULTS

### Piezo2 knockdown exerts suppressive effect on tumor growth

**Figure 1:**
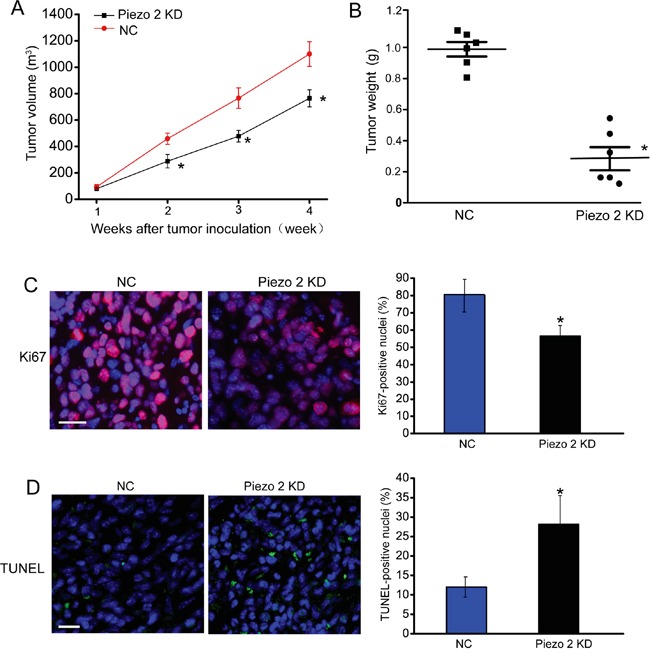
Piezo2 knockdown suppresses tumor growth **A-B.** An *in vivo* glioma model was established by injection of GL261 cells pre-transfected with scrambled shRNA (negative control, NC) or Piezo2 shRNA. Tumor volume was daily detected using a caliper and calculated by the formula: 0.52 × [width]^2^ × [length] (A, n=6 animals per group). The tumors were weighed immediately after isolation from mice. Tumor weight was plotted between the two groups (B, n=6 animals per group). Ki67 staining was conducted to detect cell proliferation. A representative image and statistical result was shown. Scar bar: 20 μm (C, n=6 animals per group). TUNEL was counterstained with DAPI (blue) for nuclei labeling. Scar bar: 20 μm. The number of Ki67-positive or TUNEL-positive cells was normalized to total nuclei number. * *P*<0.05, Piezo2 shRNA group versus NC group (D, n=6 animals per group). All data were from three independent experiments.

We first determined whether Piezo2 knockdown affects tumor growth *in vivo*. GL261 glioma cells transfected with scrambled shRNA or Piezo2 shRNA were injected subcutaneously into the right flank of nude mice. Western blot analysis revealed that Piezo2 expression was significantly reduced by Piezo2 shRNA transfection in GL261 cells (Figure S1). Tumor size was significantly reduced in Piezo2 shRNA transfected-group compared to scrambled shRNA transfected-group 2-week after tumor inoculation. At the end of experiment, tumor volume was reduced by 46% (*P*<0.01) in Piezo2 knockdown group compared to the control group (Figure 1A), which was consistent with the reduction of tumor weight (Figure 1B).

To determine the effect of Piezo2 knockdown on tumor growth, we histologically examined the end-point tumors (day 28 after implantation). As tumor growth is the net result of tumor cell proliferation and apoptosis [14], we detected tumor cell proliferation by Ki67 staining on tumor tissue sections. As shown in Figure 1C, Piezo2 knockdown resulted in a significant decrease in proliferating Ki67-positive cells. By using TUNEL cell death detection assay, we found that tumor grown in a Piezo2-knockdown environment had more TUNEL-positive apoptotic cells (Figure 1D). These results indicate that Piezo2 knockdown leads to an unfavorable environment for tumor growth by promoting tumor cell death and reducing tumor cell proliferation.

### Piezo2 is localized in tumor endothelial cells, and regulates tumor vascular architecture

**Figure 2:**
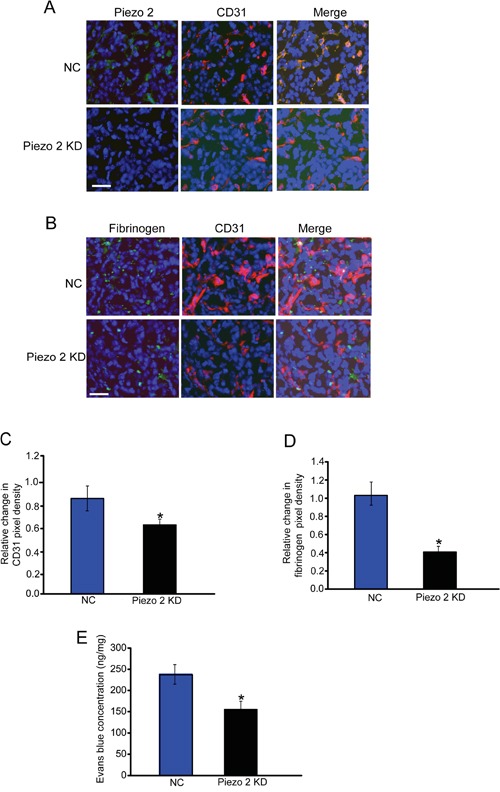
Piezo2 is localized in tumor endothelial cells, and regulates tumor angiogenesis and vascular leakage **A.** An *in vivo* glioma model was established by injection of GL261 cells pre-transfected with scrambled shRNA (negative control, NC) or Piezo2 shRNA. Tumors were excised on day 14 after implantation. Sections were double-labeled for Piezo2 (green) and vascular endothelium (CD31, red), and counterstained with DAPI (blue; n=6 animals per group). Scar bar: 20 μm. **B-D.** Excised tumors were also stained using CD31 (red) and fibrinogen (green) antibody, and then counterstained with DAPI (blue). Scar bar: 20 μm. To quantify angiogenic areas in tumor tissue, CD31 staining was quantified relative to the total pixel density (C, n=6 animals per group). Fibrinogen deposition was normalized to total CD31-positive pixel density (D, n=6 animals per group). The data was shown as relative change compared with NC group. **E.** Evans blue (30 mg/kg) was injected through the tail vein and circulated for 30 min. Tumors were excised and Evans blue concentration was quantified (n=6 animals per group). *P<0.05, Piezo2 shRNA group versus NC group. All data were from three independent experiments.

We then conducted immunofluorescent staining to detect Piezo2 expression distribution. Piezo2 (green fluorescence) was found to be mainly expressed in tumor endothelial cells (labeled by CD31, red fluorescence) in wild-type mice but not Piezo2 knockdown mice (Figure 2A). We then examined the effect of Piezo2 knockdown on tumor vascular architecture. Similar to these previous reports in many solid tumors, tumor tissues from the mice implanted with wild-type GL261 cells had increased vascular density (CD31-positive staining) and vascular leakage (fibrinogen deposition) (Figure 2B-2D). Tumor tissues from the mice implanted with Piezo2 knockdown GL261 cells showed decreased tumor vascular density and vascular leakage (Figure 2B-2D). These results suggest that Piezo2 regulates tumor angiogenesis, vascular leakage, and tumor growth. We also infused Evans blue intravenously into mice bearing equal-sized tumors to determine the effect of Piezo2 knockdown on tumor vascular permeability. We showed that Piezo2 knockdown tumor had less vascular permeability compared with the control group (Figure 2E). This result was in line with decreased fibrinogen deposition in the end-point tumors with Piezo2 knockdown.

### Piezo2 knockdown affects angiogenesis and vascular permeability *in vivo*

**Figure 3:**
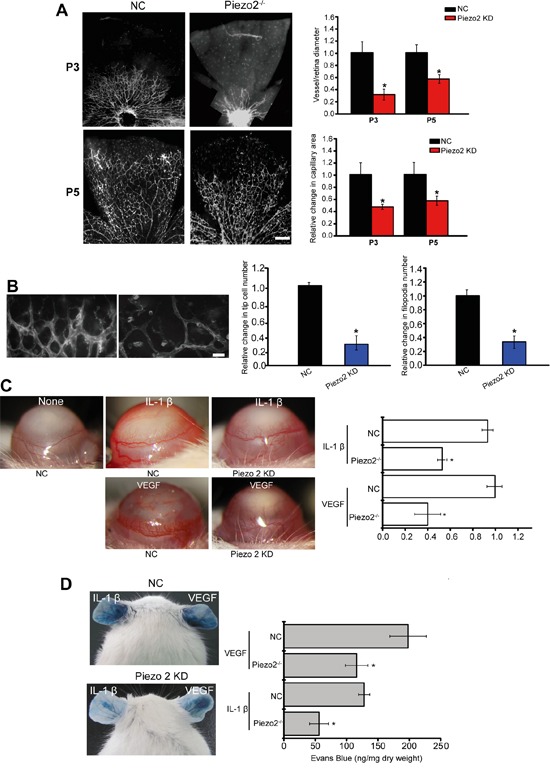
Piezo2 knockdown affects angiogenesis and vascular permeability *in vivo* **A.** The retinas of C57BL/6 mice were injected with scrambled shRNA (Negative control, NC) or Piezo2 shRNA at P1 and P3 (n=5 animals per group). Isolectin B4 staining and quantification of vascuarization was conducted at P3 and P5. Scale bar, 200 μm. **B.** Isolectin B4 stained tip cells and filopodia at P5 and quantification of tip cells and filopodia numbers was conducted (n=5 animals per group). Scale bar, 50 μm. **C.** Hydron pellets containing VEGF (50 ng) or IL-1β (10 ng) were implanted into the corneas of C57BL/6 mice (Piezo2 knockdown group and NC group). On day 5, corneal neovasculature was monitored by slit lamp and neovasculature area was quantified (n=6 animals per group, *P<0.05, Piezo2 knockdown group versus NC group). (C) VEGF (30 ng) or IL-1β (10 ng) was administered to C57BL/6 mice intradermally on the ear, and then injected with Piezo2 shRNA and scrambled shRNA (NC). Evans blue dye was injected and circulated for 30 min. Evans blue was extracted and the concentration was determined. A representative image was shown (n=6 animals per group, *P<0.05, Piezo2 knockdown group versus NC group). All data were from three independent experiments.

Primary superficial vascular network of retina is established within 7 days. The established vascular plexus then undergoes remodeling to complete vascular network. Thus, retinal vascular is a good model system for angiogenesis study [15]. We employed retinal vasculature system to define the role of Piezo2 in vascular architecture. Piezo2 knockdown displayed a significant reduction in the radial extension of vascular plexus from the optic nerve to the periphery at P3 and P5, as well as less branch points (Figure 3A). We also found that the delay in retinal angiogenesis in Piezo2 knockdown retina with fewer tip cells and filopodia compared with the control group (Figure 3B).

Tumor angiogenesis is a consequence of complex interactions affected by a set of growth factors and cytokines [16]. We further employed corneal neovascularization model to investigate the role of Piezo2 in VEGF- or IL-1β-induced angiogenesis. Hydron pellets containing VEGF or IL-1β were implanted into corneas. Corneal neovasculature extended from the limbus toward the pellets in WT and Piezo2 knockdown animals, but responses to both cytokines were decreased in corneas of Piezo2 knockdown mice (Figure 3C).

To determine the role of Piezo2 in endothelial barrier function, VEGF- and IL-1β-mediated Evans blue dye extravasation was detected in a modified Miles assay. Local VEGF or IL-1β administration resulted in a significant increase in dye extravasation, whereas Piezo2 knockdown decreased VEGF- and IL-1β-mediated dye extravasation (Figure 3D). These results suggest that Piezo2 signaling dysfunction could affect vascular leakage.

### Piezo2 knockdown affects endothelial cell and tumor cell function *in vitro*

**Figure 4:**
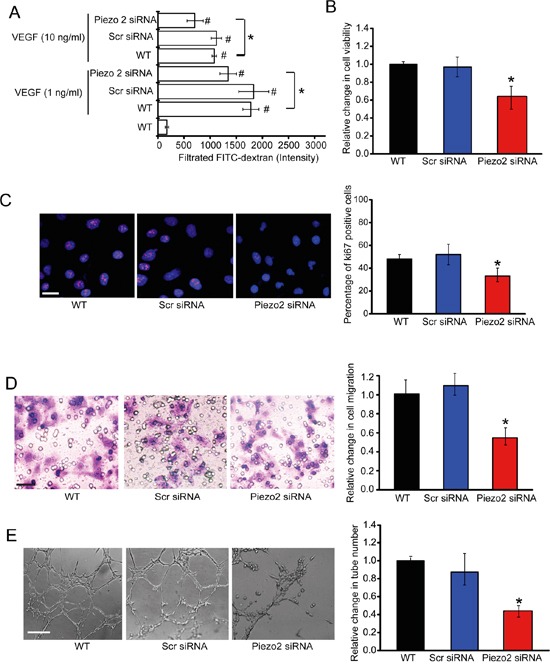
Piezo2 knockdown affects endothelial cell and tumor cell function *in vitro* **A.** Confluent monolayers of HUVECs were transfected with scrambled siRNA (Scr), Piezo2 siRNA, or left untreated (WT) for 24 h, and stimulated with VEGF (1 ng/ml or 10 ng/ml) for 30 min. After VEGF stimulation, 1% FITC-dextran was added on the upper chamber and incubated for 10 min. The amount of FITC-dextran fluxed into the lower chamber was detected (n=4; ^#^ *P*<0.05 versus WT group without VEGF treatment and ^*^ *P*<0.05, WT group +VEGF versus Piezo2 knockdown group+VEGF). **B-E.** HUVECs were transfected with Scr siRNA, Piezo2 siRNA, or left untreated (WT) for 48 h. Cell viability was detected using MTT method (B, n=4). Ki67 immunofluorescence staining was conducted to detect HUVEC proliferation and quantitative analysis. Scale bar, 20 μm (C, n=4). Transwell assays were conducted to detect tumor cell migration. Scale bar, 50 μm (D, n=4). HUVECs were transfected with scr siRNA, Piezo2 siRNA, or left untreated (WT), and cultured in 24-well plates coated with matrigel. Representative images of tube formation, tube formation number, and quantitative analysis result was shown. Scale bar, 50 μm (E, n=4). All data were from three independent experiments.

To determine whether Peizo2 knockdown affected endothelial cell junction, we performed an *In vitro* permeability assay. Confluent monolayers of HUVECs plated into trans-well plates had minimal FITC-dextran flux (Size, 70 kD) across the monolayer under non-stimulated condition, whereas administration of VEGF (10 ng/ml) greatly increased FITC-dextran flux into the lower chamber. By contrast, Piezo2 knockdown reduced VEGF-induced barrier dysfunction (Figure 4A).

One possible mechanism which Piezo2 knockdown may inhibit tumor growth is by direct anti-angiogenic effects (i.e., by inhibiting endothelial cell proliferation, migration, and tube formation). MTT assays showed that Piezo2 knockdown significantly decreased HUVECs cell viability (Figure 4B). Ki67 immunofluorescence staining showed that Piezo2 knockdown decreased the proliferation ability of HUVECs (Figure 4C). Transwell assays were also conducted to determine the effect of Piezo2 knockdown on HUVECs migration. Piezo2 knockdown significantly decreased HUVECs migration compared with scrambled siRNA-transfected group (Figure 4D). Piezo2 knockdown also decreased tube formation of HUVECs compared with the control group (Figure 4E). *In vitro* co-culture system was also conducted to determine the effect of Piezo2 knockdown in endothelial cells on tumor cell function. We showed that Piezo2 knockdown in endothelial cells decreased tumor cell proliferation, migration, and invasion of tumor cells (Figure S2).

### Piezo2 knockdown affects intracellular [Ca2+] and Wnt11 expression

**Figure 5:**
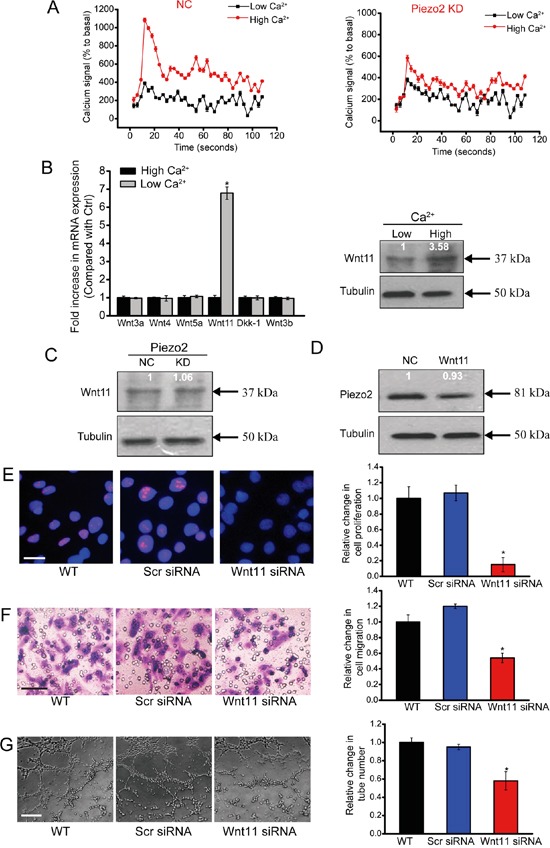
Piezo2 knockdown affects intracellular [Ca2+] and Wnt11 expression **A.** Detecton of intracellular calcium in HUVECs transfected with Piezo2 siRNA (KD) or scrambled siRNA (NC). After 5 seconds, 0.1 mM Ca^2+^ (low Ca^2+^) or 2.0 mM Ca^2+^ (high Ca^2+^) was added to the cells. Responses were normalized by defining the first value as 100% (n=4). **B.** HUVECs were incubated in medium containing 0.1 mM Ca^2+^ (low Ca^2+^) and 2.0 mM Ca^2+^ (high Ca^2+^) for 24 h. mRNA expression of Wnt3a, Wnt3b, Wnt4, Wnt5a, Wnt11, and Dkk-1 was determined by qRT-PCRs. The values were normalized to Tubulin mRNA. Results were shown as relative change compared with the cells grown at low Ca^2+^ (n=4). Protein expression of Wnt11 was detected by western blot analysis of aliquots from 2-day culture supernatants. Equal amounts of total protein were applied in each lane. **C.** Western blot analysis of Wnt11 in the supernatants of HUVECs transfected with Piezo2 siRNA (KD) or scrambled siRNA (NC) on day 2 after incubation in high ex[Ca^2+^] (n=4). **D.** Western blot analysis of Piezo2 expression in extracts from HUVECs incubated for 2 days with and without (NC) 200 ng/ml Wnt11. Cellular tubulin was detected as the loading control (n=4). **E-G.** HUVECs were transfected with scrambled siRNA (Scr), Wnt11 siRNA, or left untreated (WT) for 48 h. Ki67 staining was conducted to detect HUVEC proliferation and quantitative analysis. Scale bar, 20 μm. (E, n=4). Transwell assays were conducted to detect tumor cell migration. Scale bar, 50 μm (F, n=4). HUVECs were cultured in 24-well plates coated with matrigel. Representative images of tube formation, a tube formation number, and quantitative analysis result was shown. Scale bar, 50 μm (G, n=4). All data were from three independent experiments.

Piezo2 is a new family of cation-permeable and directly mechanically activated ion channel with a selectivity sequence of Ca^2 +^ > K^+^ > Na^+^ > Mg^2+^ [7]. Ca^2+^ signaling in the endothelium is fundamental for vascular tone and arterial blood pressure regulation [17]. We thus determined whether Piezo2 knockdown affected intracellular [Ca^2+^] (in[Ca^2+^]). HUVECs were transfected with Piezo2 siRNA to down-regulate Piezo2 level. High extracellular [Ca^2+^] (ex[Ca^2+^]) caused an obvious increase in in[Ca^2+^] followed by a rapid decline and sustained increase of in[Ca^2+^] compared with cells incubated in low ex[Ca^2+^]. In Piezo2-konckdown HUVECs, both the ex[Ca^2+^]-induced initial peak and sustained increase of in[Ca^2+^] were significantly reduced (Figure 5A), suggesting that Piezo2 is involved in [Ca^2+^] regulation in HUVECs.

Accumulating data indicates the importance of Wnt signaling in endothelial cell proliferation, migration and survival [18]. We then determined whether increased [Ca^2+^] regulates Wnt signaling activity. Incubation of HUVECs with high ex[Ca^2+^] did not induce or affect the transcription of Wnt3a, Wnt3b, Wnt5a, Wnt4, and Dkk-1 in HUVECs, but significantly increased Wnt11 mRNA expression and protein secretion compared with cells grown at low ex[Ca^2+^] (Figure 5B).

High ex[Ca^2+^]-induced Wnt11 up-regulation was significantly blocked upon Piezo2 knockdown in HUVECs (Figure 5C). By contrast, basal production of Piezo2 in HUVECs was not affected with or without Wnt11 treatment (Figure 5D). These results suggest that Wnt11 biosynthesis is dependent on Piezo2 activity, while Piezo2 expression is independent of Wnt11 activity in HUVECs.

We then determined whether Wnt11 affects the angiogenic activity of endothelial cells (eg, proliferation, migration, and tube formation). Wnt11 knockdown significantly reduced the proliferation (Figure 5E), migration (Figure 5F), and tube formation (Figure 5G) ability of endothelial cells, suggesting a key role of Wnt11 in angiogenic activity of endothelial cells.

### β-catenin is involved in Wnt11/high ex[Ca2+]-modulated angiogenic activity of endothelial cells

Wnt/β-catenin signaling has been reported to regulate tumor angiogenesis [19]. We further investigated whether Wnt11/high ex[Ca^2+^] treatment could regulate Wnt/β-catenin signaling. Wnt/β-catenin signaling activation is characterized by cytoplasmatic β-catenin stabilization, β-catenin nuclear translocation, and increased expression of β-catenin target genes [20]. Thus, we investigated each of these features experimentally.

**Figure 6:**
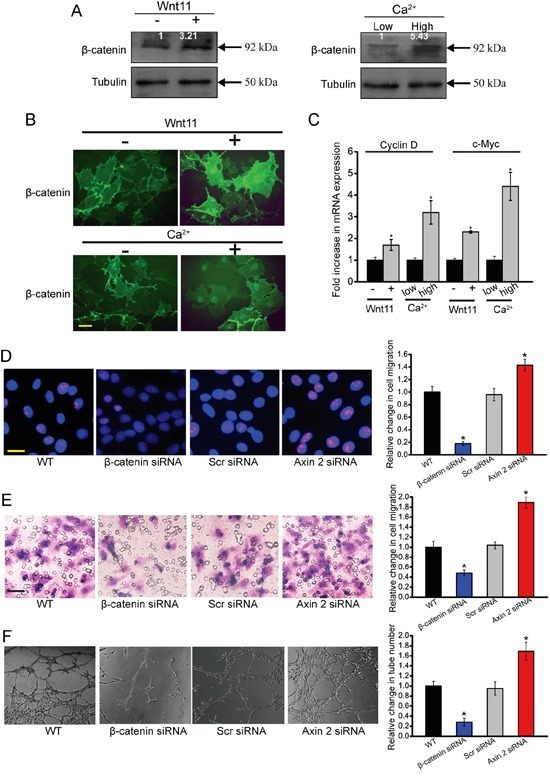
β-catenin is involved in Wnt11/high ex[Ca2+]-modulated angiogenic activity of endothelial cells **A-C.** HUVECs were incubated with or without recombinant Wnt11 (150 ng/ml) as well as medium containing 0.1 mM Ca^2+^ (low Ca^2+^) and 2.0 mM Ca^2+^ (high Ca^2+^). β-catenin expression was determined by western blot analysis of cell extracts after 24 h cultivation. For densitometry, β-catenin amount in cells grown without recombinant Wnt11 and at low ex[Ca^2+^] was set as 1. Tubulin was detected as the control (A, n=4). Immunofluoresence assays were conducted to detect β-catenin distribution in HUVECs after 24 h cultivation. Scale bar, 20 μm (B, n=4). The transcription levels of Wnt/β-catenin target genes, cyclin D1 and c-myc, were detected using qRT-PCRs. The values were normalized to Tubulin mRNA. The results showed mRNA expression change relative to cells grown without exogenous Wnt11 and at low ex[Ca^2+^] (C, n=4). **D-F.** HUVECs were transfected with scrambled siRNA (Scr), β-catenin siRNA, axin 2 siRNA, or left untreated (WT) for 48 h. Ki67 staining was conducted to detect HUVEC proliferation. Scale bar, 20 μm (D, n=4). Transwell assays were conducted to detect tumor cell migration. Scale bar, 50 μm (E, n=4). HUVECs were cultured in 24-well plates coated with matrigel. Representative images of tube formation, tube formation number, and quantitative analysis data was shown. Scale bar, 50 μm (F, n=4). “^*^” indicated significant difference compared with the corresponding control group. All data were from three independent experiments.

Western blots analysis revealed that β-catenin protein levels were elevated in HUVECs treated with exogenous Wnt11 or high ex[Ca^2+^] compared with control cells (Figure 6A). Immunofluoresence assay showed that bothexogenous Wnt11 and high ex[Ca^2+^] treatment contributed to β-catenin nuclear translocation (Figure 6B). To further determine the stimulatory effect of Wnt11 and high ex[Ca^2+^] on β-catenin-dependent signaling, we performed qRT-PCR analysis of Wnt/β-catenin target genes, cyclin D1 and c-Myc. Both Wnt11 treatment and high ex[Ca^2+^] resulted in a significant increase in the transcription of cyclin D1 and c-Myc (Figure 6C). Collectively, the above-mentioned results show that Wnt11 and high ex [Ca^2+^] treatment could activate Wnt/β-catenin signaling.

To determine the involvement of β-catenin pathway in endothelial cell function, we employed β-catenin siRNA to down-regulate β-catenin level. β-catenin-knockdown HUVECs showed a significant decrease in β-catenin transcription, protein production, and target gene expression (Figure S3). β-catenin-knockdown HUVECs had decreased cell proliferation, migration, and tube formation (Figure 6D-6F). We also increased the intracellular stability of β-catenin by siRNA-mediated knockdown of axin 2, a cytoplasmatic protein that has a key role in the degradation and activity of β-catenin [20]. Measurement of increased levels of β-catenin target gene, cyclin D1, confirmed that the axin 2 knockdown in HUVECs was functional (Figure S4). Axin 2 knockdown resulted in a significant increase in cell proliferation, cell migration, and tube formation (Figure 6D-6F). Collectively, these results provide evidence that β-catenin signaling plays a key role in endothelial cell function.

### Piezo2 regulates the angiogenic activity of endothelial cells via β-catenin signaling

**Figure 7:**
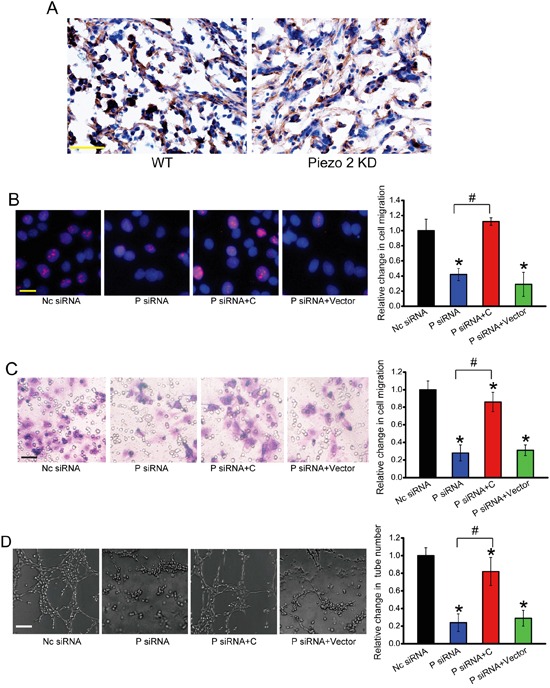
Piezo2 regulates the angiogenic activity of endothelial cells via β-catenin signaling **A.** β-catenin expression was detected by western blots in Piezo2 knockdown tumors and control tumors. Scar bar, 50 μm. n=6 animals per group. **B-D.** HUVECs were transfected with scrambled siRNA (Nc), Piezo2 siRNA, Piezo2 siRNA+β-catenin-pcDNA 3.0 plasmid, or Piezo2 siRNA+ pcDNA 3.0 vector for 48 h. Ki67 staining and quantitative analysis was conducted to detect HUVEC proliferation. Scale bar, 20 μm (B, n=4). Transwell assays were conducted to detect tumor cell migration. Scale bar, 50 μm (C, n=4). HUVECs were cultured in 24-well plates coated with matrigel. Representative images of tube formation, tube formation number, and quantitative analysis result was shown. Scale bar, 50 μm (D, n=4). “^*^” indicated significant difference compared with Nc group. “^#^” indicated significant difference between the marked groups. All data were from three independent experiments.

To determine whether Peizo2 knockdown affects β-catenin signaling *in vivo*, we performed immunohistochemical analysis from Piezo2 knockdown tumors and control tumors. Piezo2 knockdown could significantly suppress β-catenin nucleus translocation compared with the control group (Figure 7A). We further determined whether Piezo2-β-catenin crosstalk is involved in the regulation of angiogenic activity of endothelial cells. Ki67 staining revealed that Piezo2 knockdown obviously inhibited the proliferative ability of HUVECs, whereas β-catenin overexpression could accelerate the proliferation of HUVECs (Figure 7B). β-catenin overexpression could partially reverse Piezo2 knockdown-mediated inhibitory effect on cell migration and tuber formation of HUVECs (Figure 7C and 7D). Collectively, these results suggest that Piezo2 regulates the angiogenic activity of endothelial cells via β-catenin signaling.

## DISCUSSION

In the present study, we revealed that Piezo2 was localized in the endothelial cells in tumor vasculature and its knockdown inhibited tumor angiogenesis, vascular leakage, and tumorigenesis. Furthermore, Piezo2 knockdown led to abnormal intracellular [Ca^2+^], Wnt11/β-catenin signaling reduction, and altered angiogenic activity of endothelial cells. Thus, Piezo2 is promising target for anti-angiogenic therapy.

Piezo ion channels are essential for mechanical responses. Both piezo1 and piezo2 channels are cation selective mechanical channels with a selectivity sequence of Ca^2 +^ > K^+^ > Na^+^ > Mg^2 +^ [21, 22]. In primary articular chondrocytes, expression of Piezo1 and −2 induces Ca^2+^ signals and electrical currents. Moreover, mechanically evoked Ca^2+^ transients produced by atomic force microscopy are inhibited by GsMTx4, a Piezo-blocking peptide, and by Piezo1- or Piezo2-specific siRNA [23]. Previous studies have revealed that Piezo2 is expressed in subsets of myelinated and unmyelinated sensory neurons. Piezo2 constitutive knockout mice die at birth [11]. Multifaceted biological actions of Piezo2 are still unknown. We revealed that Piezo2 knockdown could affect intracellular [Ca^2+^]. Ca^2+^ signaling in the endothelium is fundamental for the regulation of vascular tone, arterial blood pressure, and angiogenesis [17, 24]. Piezo2 mediates Ca^2+^ entry following receptor stimulation and mechanical stimulation of angiogenic activity of endothelial cells. Piezo2 intervene could suppresses tumor expansion by a concomitant decrease in tumor vascularity.

It is widely accepted that solid tumors require angiogenesis to grow beyond a certain size. This process involves several inflammatory factors and cell factors such as VEGF, fibroblast growth factor, angiopoietin, IL-1, and tumor necrosis factor [3]. Piezo2 knockdown decreased VEGF- or IL-1β-mediated pathological angiogenesis, implying that Piezo2 plays an anti-angiogenic role during tumor growth. Tumor vasculature is usually hyperpermeable to macromolecules compared to normal vasculature. Anti-permeability peptide, cavtratin and TNP-470, have been shown to suppress vascular leakage and tumor growth [25, 26]. VEGF is a prominent cytokine responsible for the hyperpermeable state of tumor vessels [27, 28]. The modified Miles assay revealed that Piezo2 knockdown decreased VEGF- and IL-1β-mediated vascular leak, suggesting that Piezo2 plays an anti-permeability role during tumor angiogenesis. The reduction in hyper permeability may be due to decreased angiogenesis. Thus, Piezo2 knockdown mice developed less permeable and smaller tumors compared with wild-type mice. Given the critical role of Piezo2 in tumor angiogenesis and vascular hyperpermeability, it is not surprised that Piezo2 is a potential target for anti-angiogenic therapy during tumor treatment.

Wnt/β-catenin pathway is an important signaling pathway that directs cell proliferation, self-renewal, differentiation, and tissue homeostasis [20, 29]. Deregulation of Wnt/β-catenin pathway contributes to human degenerative disease and tumorigenesis. Wnt proteins represent a family of secreted, lipid-modified glycoproteins. Upon binding to certain members of the Frizzled family of Wnt receptors such as Wnt 4, Wnt 5A or Wnt 11, they are able to elicit an intracellular release of calcium ions [30]. Wnt/Ca^2+^ pathway are also known to control cell proliferation and migration, although whether they promote or suppress cell motility seems to depend on cell type [31]. Piezo2 knockdown affected intracellular Ca^2+^, and significantly reduced the biosynthesis and secretion of Wnt11. Wnt11 up-regulation could trigger cytoplasmic release of Ca^2+^ and calcium related signaling. Previous studies show that noncanonical Wnt11 inhibits hepatocellular carcinoma cell proliferation and migration [32]. Canonical Wnt11 promotes neuroendocrine-like differentiation, survival and migration of prostate cancer cells [33], and is induced by estrogen-related receptor α and β-catenin and acts in an autocrine manner to increase cancer cell migration [34]. We showed that Wnt11 promoted the proliferation, migration, and tube formation of endothelial cells. We speculated that Wnt11/β-catenin may be involved in angiogenic activity of endothelial cells.

Previous studies show that Wnt11 activates β-catenin pathway activity depending on cell types and receptor context. Wnt11 acts in an autocrine manner to alter cell migration, cell proliferation, patterning N-cadherin and β-catenin expression, or axin expression [34–36]. Exogenous application of Wnt11 enhanced the stability, nucleus translocation and promoter activity of β-catenin, suggesting that Wnt11 is a positive regulator of β-catenin-dependent signaling in endothelial cells. Altered Wnt/β-catenin signaling in the endothelium contribute to pathological angiogenesis [37]. Thus, continued progress in this field holds the potential to identify novel therapeutics for the treatment of pathological angiogenesis.

In conclusion, we demonstrated that Piezo2 knockdown led to tumor growth inhibition, reduced vascular density and vascular hyperpermeability. Extracellular Ca^2+^ could increase free intracellular Ca^2+^ and up-regulate the expression and secretion of Wnt11 in endothelial cells, while this increase was interrupted in Piezo2 knockdown cells. Wnt11 acted as an autocrine stimulus by increasing β-catenin stability, β-catenin nucleus translocation, and β-catenin signaling activity that potentiated the angiogenic activity of endothelial cells (Figure S5). Piezo2 inhibitor may be developed as a promising drug for anti-angiogenic therapy.

## MATERIALS AND METHODS

### Cell culture and transfection

GL261 cells and HUVECs were maintained in the Dulbecco's Modified Eagle's medium (DMEM) supplemented with 10% fetal bovine serum (FBS) in a humidified incubator with 5% CO_2_ at 37°C. Cell transfection was conducted using lipofectamine 2000 (Life Technologies) according to the manufacturer's instruction.

### Tumor experiment

All animal experiments were performed in accordance with the Chinese Legislation on the Protections of Animals and the Guide for the Care and Use of Laboratory Animals with permission of Nanjing Medical University (Nanjing, China). C57BL/6 mice were anesthetized by i.p. injection of Ketamine/Xylazine. An *in vivo* glioma model was established by injection of GL261 cells pre-transfected with scrambled shRNA or Piezo2 shRNA. About 10^6^ cells were implanted subcutaneously into the flanks of 8-week-old female nude C57BL/6 mice. Tumor volume was determined daily using a caliper and calculated by the formula: 0.52× [width]^2^× [length].

### Tumor permeability and modified miles assay

For tumor permeability assay, 14 days after glioma tumor implantation, Evans blue (30 mg/kg) was injected through the tail vein and circulated for 30 min. For the modified Miles assay, 12-week-old male C57BL/6 mice were used. After treatment with test agents, animals were anesthetized with ketamine/xylazine. VEGF, IL-1β, or saline solution was then injected intradermally into the dorsal ear skin before Evans blue dye (30 mg/kg) injection and circulation for 30 min. For both assays, animals were killed and perfused with 0.5% paraformaldehyde before tissues were excised, dried, and weighed. Evans blue dye was extracted in formamide, and its content was quantified by reading at 610 nm in a microplate reader (Molecular Devices).

### Quantification of corneal angiogenesis

Hydron pellets containing VEGF or IL-1Δ were prepared and implanted in corneas of 12-week-old male C57BL/6 mice. On day 6, the mice were anesthetized and corneal vessels were photographed. Areas of corneal neovascularization were analyzed using the Image J software and expressed in mm^2^. The neovascular area was determined by subtracting the non-stimulated vascular area from the vascular area.

### Immunofluorescence and TUNEL assay

The cryosections were fixed in 4% paraformaldehyde for 5 min, washed 3 times in PBST and blocked with 4% bovine serum albumin (BSA) for 1 h at 4°C. The primary antibodies used included Piezo2 (1:200, Abcam), CD31 (1:1,000, BD Biosciences), and fibrinogen (1:1,000, Santa Cruz). TUNEL assay kit (Roche) was used to label apoptotic cells according to the manufacturer's instruction.

After specific treatment, HUVECs or GL261 cells were fixed with the ice-cold methanol for 10 min at −20°C. Non-specific binding was blocked with 5% BSA for 30 min. These cells were incubated with the primary antibody (Ki67, 1:200, Abcam) overnight at 4°C, and then incubated with the secondary antibody conjugated with Alexa Fluor 594 (Invitrogen) for 5 h at room temperature, followed by incubation with 4′,6-Diamidino-2-phenylindole dihydrochloride (DAPI, Sigma-Aldrich) for 10 min. The cells were observed using a fluorescence microscope.

### MTT assay

Cell viability was detected using 3-(4, 5-dimethylthiazol-2-yl)-2, 5- diphenyl-tetrazolium-bromide assay (MTT). Briefly, cells were plated at a density of 2×10^4^ cells per well in 96-well plates. After specific treatment, they were incubated with MTT (0.5 mg/ml) at 37°C for 3 h. Finally, 100 mM DMSO solution was added to dissolve the formazan crystals after the medium removal. The absorbance was detected at 570 nm wavelength using a microplate reader (Molecular Devices).

### Cell invasion and migration assay

24-well plates with 8-μm micropore inserts were used for cell invasion and migration assay. For cell invasion assay, the top side of the insert was coated with Matrigel (BD Biosciences). After specific treatment, GL261 cells were placed into the upper well, cultured for 24 h and allowed to invade into the Matrigel layer. For migration assay, GL261 cells or HUVECs were placed into the upper well, cultured for 24 h, and allowed to invade through the transwell plate. The cells on the inserts were fixed with 3% paraformaldehyde, stained with crystal violet, and counted using a light microscope. Invasion or migration was shown as fold change of the number of cells invading or migrating through the transwell plate.

### Measurement of endothelial permeability

Confluent monolayers of HUVECs formed on the transwell inserts with 8 μm pores (Falcon; BD). Test agents were exposed to the upper and lower chamber, and then VEGF was added to the upper chamber for 15 min. FITC-dextran (1%) was added to the upper chamber and the entire chamber was incubated for 15 min. Aliquots (100 μl) were then retrieved from the lower chamber and FITC concentration was detected using a fluorescence spectrophotometer.

### Immunohistochemistry

Tissue sections (20 μm) were incubated with the primary antibody (β-catenin, 1:200) for 24 h and washed three times using PBS buffer (pH 7.2). Sections were then incubated with the biotinylated secondary antibodies (GE Healthcare) and streptavidin HRP (horseradish peroxidase, Dako) for 30 min. Sections were washed in PBST (3 × 10 min), rinsed in 0.1 M Tris-HCl (pH 7.5) for 5 min. The reaction products were visualized using 0.1 M Tris-HCl (pH 7.5) containing 0.05% 3/3′-diaminobenzidine tetrahydrochloride and 0.003% hydrogen peroxide. These sections were then counterstained with hematoxylin.

### Tube formation assay

The basement membrane matrix (BD Biosciences) was placed into the well of 24-well plate, and hardened for 30 min at 37°C. 3× 10^4^ HUVECs were seeded on each well for tube formation at 37°C for 24 h. The tube formation was observed using Olympus IX-73 microscope.

### Western blot

Protein extraction from HUVECs, and equal amount of protein was loaded per lane for sodium dodecyl sulfate-polyacrylamide gel electrophoresis (SDS-PAGE). The blotted membranes were incubated overnight with polyclonal antibodies against Wnt11a (Abcam, 1:1,000), Piezo2 (Abcam, 1:1,000), β-catenin (Cell Signaling, 1:1,000), Tubulin (Cell Signaling, 1:1,000) diluted in Tris-buffered saline containing 0.1% Tween-20 (Tris-buffered saline-Tween-buffer). The membranes were washed and then incubated with the secondary antibody (Cell Signaling) conjugated with peroxidase (1:5,000) for 3 h. Bound antibodies were detected using the enhanced chemiluminescence system (GE Healthcare).

### Quantitative reverse transcription PCR (qRT-PCR)

Total RNAs were extracted using TRIzol reagent (Invitrogen). cDNAs were synthesized using a High Capacity cDNA Reverse Transcription Kit (Applied Biosystems). qRT-PCRs were conducted using the Power SYBRs Green PCR Master Mix (Applied Biosystems) on the StepOne Plus™ Real-Time PCR System. The PCR profile was 94°C for 10 min, and 42 cycles of 94°C for 10 s and 58°C for 15 s. PCR products were verified by melting curve analysis as well as by 2.0% agarose gel electrophoresis. Data analysis was conducted using the 2^−ΔΔCt^ method.

### Intracellular [Ca2+] detection

HUVECs were loaded with 1 μM Fluo-4/AM (Molecular Probes) for 20 min at 37°C in the dark and then washed three times. Dynamic measurements of Ca^2+^ concentrations were performed in the same plate reader (Molecular Device) using fluorescence optics and light guide with 485BP12 excitation and Em520 emission filters in cells loaded with Fluo-4/AM.

### Statistical analysis

All data was shown as means±SEM. Unless otherwise mentioned, the differences between groups were analyzed using Student's *t* test when only two groups were compared or by one-way analysis of variance (ANOVA) when more than two groups were compared. All statistical analyses were carried out using SPSS 13.0 statistical software. The Kaplan-Meier method was used to establish survival curves, and the survival differences were compared using the log-rank test. *P*< 0.05 was considered statistically significant.

## SUPPLEMENTARY FIGURES


