# Supplementary material for: Piezo2 protein: A novel regulator of tumor angiogenesis and hyperpermeability
Source: Oncotarget. 2016 Jun 17;7(28):44630–43. doi: 10.18632/oncotarget.10134 (PMC5190124; doi:10.18632/oncotarget.10134)
Supplement: Supplementary file 1 [file oncotarget-07-44630-s001.pdf]

Piezo2 protein: A novel regulator of tumor angiogenesis and hyperpermeability

SUPPLEMENTARY FIGURES

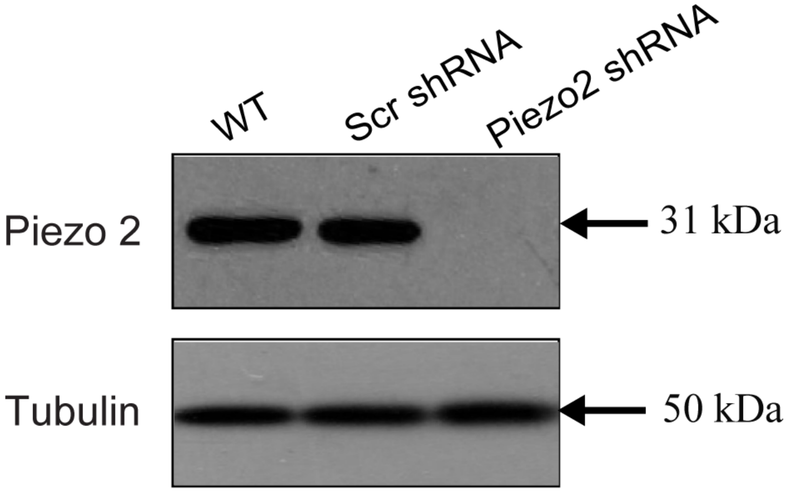

Supplementary Figure S1: Detection of Piezo 2 expression by western blots. GL261 glioma cells were transfected with scrambled shRNA (Scr), Piezo2 shRNA, or left untreated (WT) for 48 h, and then injected subcutaneously into the right flank of 8-week-old female nude C57BL/6 mice. Two weeks after injection, tumor tissue was harvested and immediately frozen in liquid nitrogen for western blot analysis of Piezo2 expression. Tubulin was detected as the internal control. n=6 animals per group. Data was from three independent experiments.

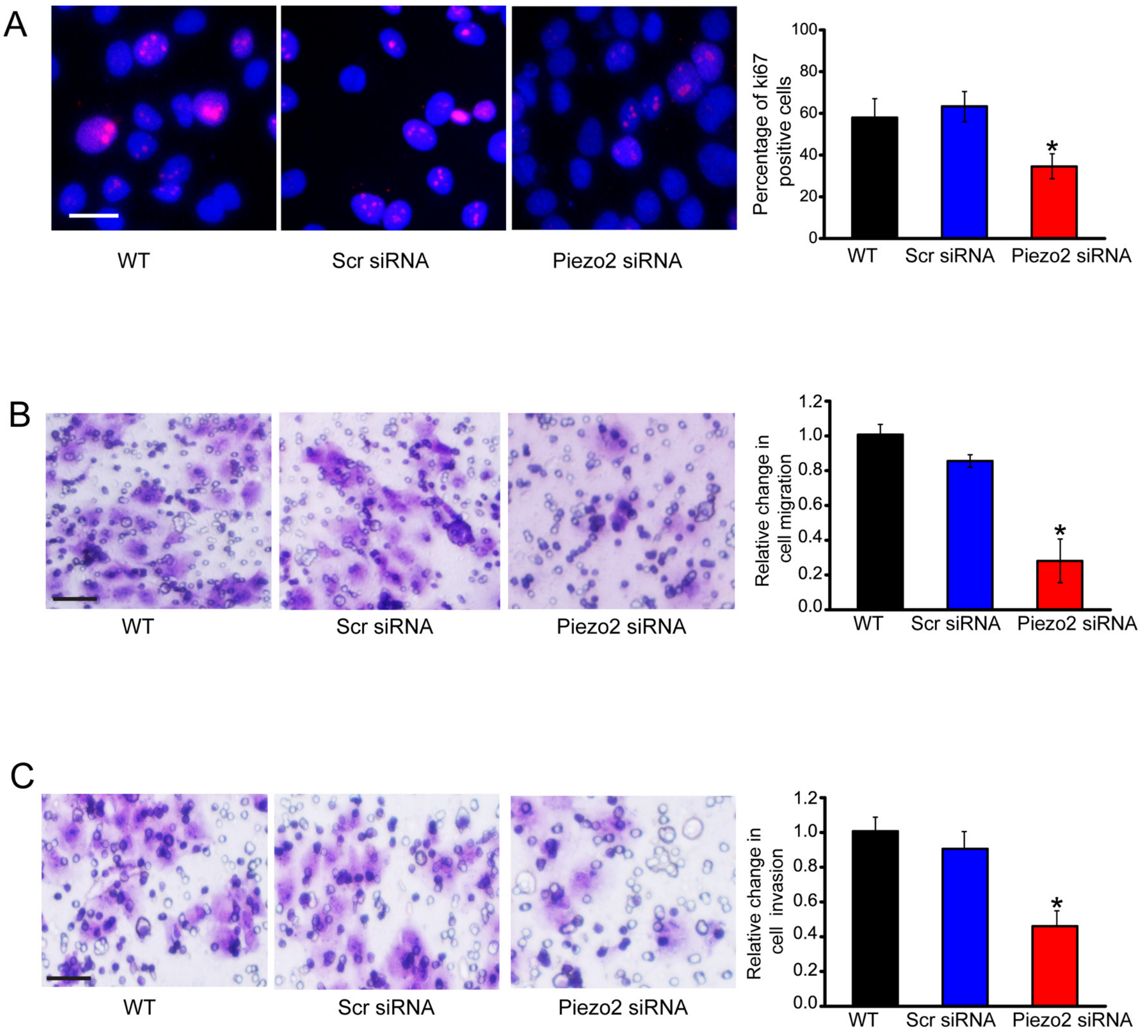

Supplementary Figure S2: Effect of Piezo2 knockdown in HUVECs on tumor cell function. HUVECs were transfected with scrambled siRNA (Scr), Piezo2 siRNA, or left untreated (WT), and then co-cultured with GL261 glioma cells for 48 h. Ki67 staining was conducted to detect tumor cell proliferation (n=4, A) Scale bar, 20 µm. Transwell assays were conducted to detect tumor cell migration and invasion (n=4, B and C). Scale bar, 50 µm. Data was from three independent experiments.

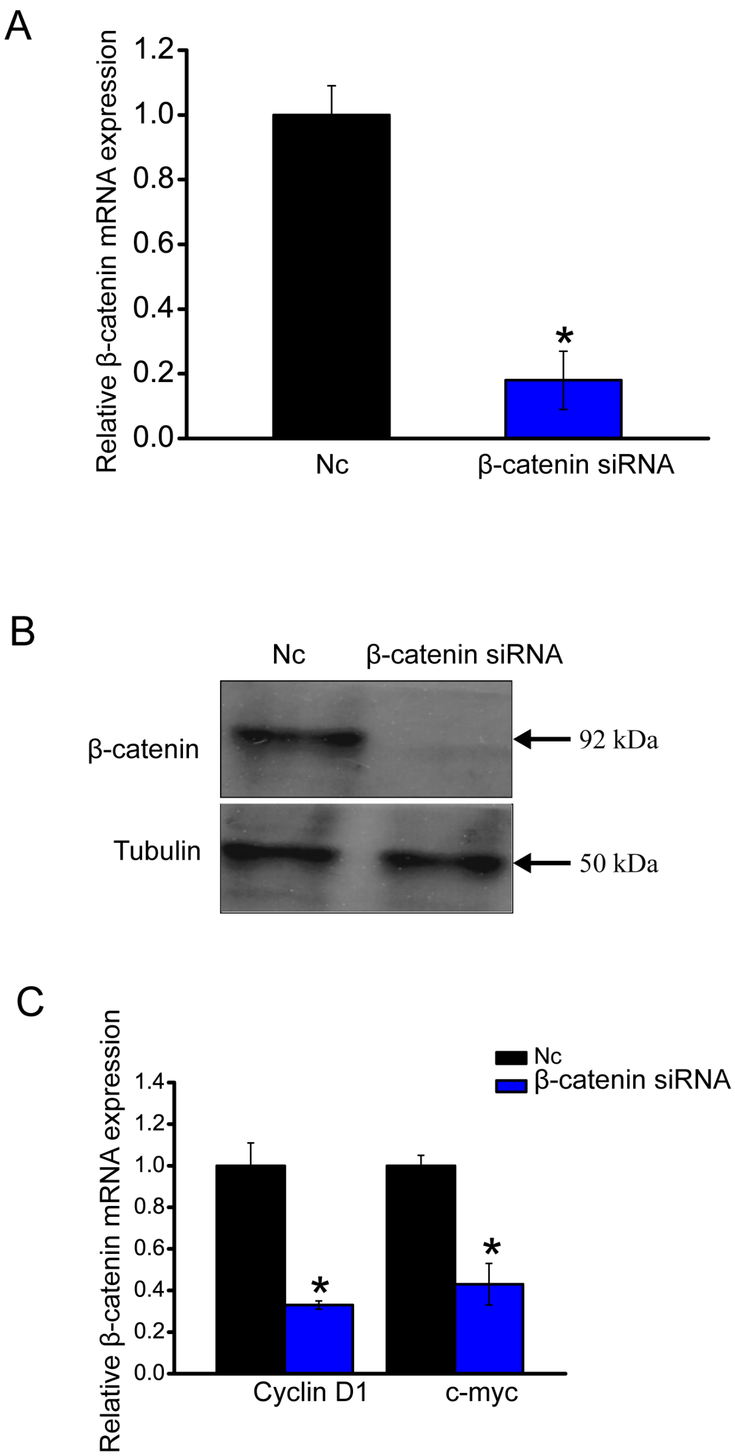

Supplementary Figure S3: Knockdown of β-catenin in HUVECs. HUVECs were transfected with β-catenin siRNA (KD) or scrambled siRNA (NC) for 24 h. β-catenin expression was determined by qRT-PCR and western blot (A and B). In addition, transcription of β-catenin target gene, cyclin D1 and c-Myc, was detected by qRT-PCR to confirm the efficacy of β-catenin knockdown in HUVECs (n = 4). *$P < 0.05$. Data was from three independent experiments.

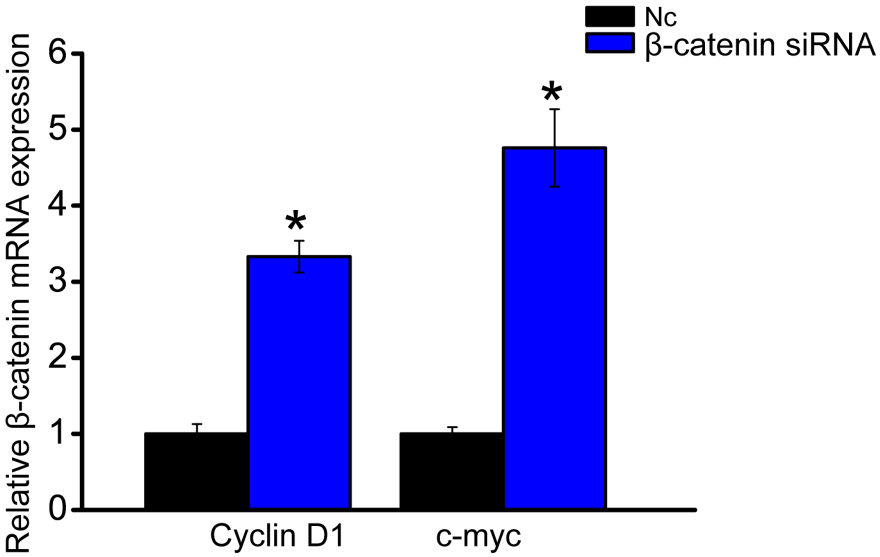

Supplementary Figure S4: Functional analysis of axin 2 knockdown in HUVECs by detecting β-catenin target gene expression. HUVECs were transfected with axin 2 siRNA (KD) or scrambled siRNA (NC). After cultivation for 24 h, the expression levels of cyclin D1 and c-Myc were detected using qRT-PCRs (n = 4). Data was from three independent experiments.

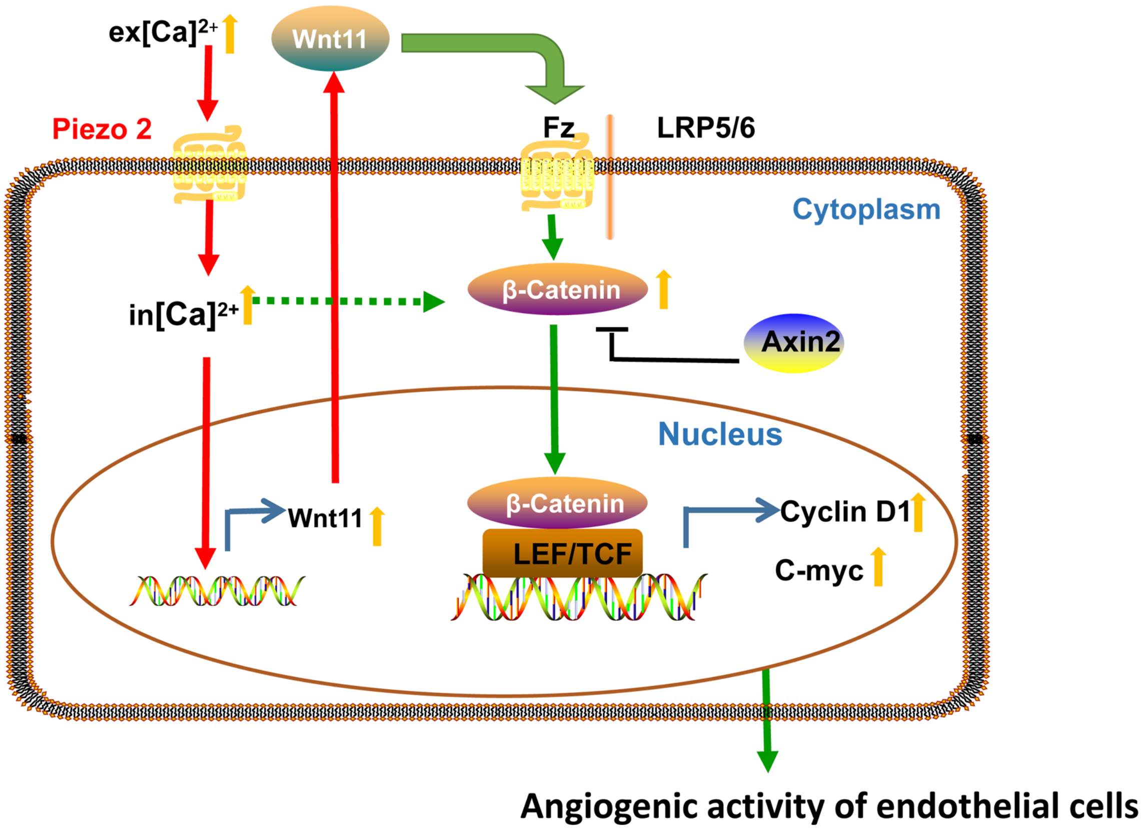

Supplementary Figure S5: Proposed model for Piezo2/Wnt11/β-catenin signaling in the regulation of angiogenic activity of endothelial cells. Elevated levels of extracellular calcium concentration (ex[Ca^{2+}]) stimulate intracellular signaling through Piezo2. This leads to an increase in free intracellular calcium (in[Ca^{2+}]). As a consequence, the expression and secretion of Wnt11 are up-regulated. Wnt11 then binds to members of the Frizzled (Fz) family of receptors and low-density lipoprotein receptor-related protein (LRP) 5/6 on the cell surface, thus activating the canonical Wnt/β-catenin signaling pathway through augmentation of β-catenin in endothelial cells. Improved interaction of nuclear β-catenin with lymphoid-enhancer-binding factor-1/T-cell factor-1 (LEF/TCF) transcription factors then leads to the up-regulation of typical target genes such as cyclin D1 and c-Myc, which regulates the angiogenic activity of endothelial cells.
